# Genome-wide analysis toward the epigenetic aetiology of myelodysplastic syndrome disease progression and pharmacoepigenomic basis of hypomethylating agents drug treatment response

**DOI:** 10.1186/s40246-023-00483-7

**Published:** 2023-04-25

**Authors:** Stavroula Siamoglou, Ruben Boers, Maria Koromina, Joachim Boers, Anna Tsironi, Theodora Chatzilygeroudi, Vasileios Lazaris, Evgenia Verigou, Alexandra Kourakli, Wilfred F. J. van IJcken, Joost Gribnau, Argiris Symeonidis, George P. Patrinos

**Affiliations:** 1 Laboratory of Pharmacogenomics and Individualized Therapy, Department of Pharmacy, University of Patras, School of Health Sciences, University Campus, 265 04 Rion, Patras, Greece; 2 Department of Developmental Biology, Erasmus Medical Center, Rotterdam, the Netherlands; 3 Hematology Division, Department of Internal Medicine, University of Patras Medical School, Patras, Greece; 4 Erasmus Medical Center, Center for Biomics, Rotterdam, The Netherlands; 5 Department of Genetics and Genomics, United Arab Emirates University, College of Medicine and Health Sciences, Al-Ain, Abu Dhabi, United Arab Emirates; 6 Zayed Center for Health Sciences, United Arab Emirates University, Al-Ain, Abu Dhabi, United Arab Emirates

**Keywords:** Myelodysplastic syndromes, Acute myelogenous leukemia, HMA treatment, Whole methylome analysis, RAEBI, RAEBII, DMRs, *PCDHG* and *ZNF*

## Abstract

Myelodysplastic syndromes (MDS) consist of a group of hematological malignancies characterized by ineffective hematopoiesis, cytogenetic abnormalities, and often a high risk of transformation to acute myeloid leukemia (AML). So far, there have been only a very limited number of studies assessing the epigenetics component contributing to the pathophysiology of these disorders, but not a single study assessing this at a genome-wide level. Here, we implemented a generic high throughput epigenomics approach, using methylated DNA sequencing (MeD-seq) of LpnPI digested fragments to identify potential epigenomic targets associated with MDS subtypes. Our results highlighted that *PCDHG* and *ZNF* gene families harbor potential epigenomic targets, which have been shown to be differentially methylated in a variety of comparisons between different MDS subtypes. Specifically, CpG islands, transcription start sites and post-transcriptional start sites within *ZNF124*, *ZNF497* and *PCDHG* family are differentially methylated with fold change above 3,5. Overall, these findings highlight important aspects of the epigenomic component of MDS syndromes pathogenesis and the pharmacoepigenomic basis to the hypomethylating agents drug treatment response, while this generic high throughput whole epigenome sequencing approach could be readily implemented to other genetic diseases with a strong epigenetic component.

**Supplementary Information:**

The online version contains supplementary material available at 10.1186/s40246-023-00483-7.

## Introduction

Myelodysplastic syndromes (MDS) represent a heterogeneous group of hematological malignancies, characterized by ineffective hematopoiesis, cytogenetic abnormalities, and a high risk of transformation to acute myeloid leukemia (AML). MDS occurs predominantly in the elderly people of approximately over 70 years of age, with the incidence rate to be about 4–5 new cases per 100.000 people per year [1–5]. There is evidence of a trend for increasing incidence the last decades and this might in part be attributed to earlier diagnosis or to increased environmental risk factors [6]. Three major components have been found to contribute to the pathogenesis of primary MDS: First, somatic variants, most commonly initially affecting spliceosomal and DNA-methylation controlling genes, named driver mutations [7], second, epigenetic modulation of crucial genes controlling growth and differentiation functions of the hematopoietic stem cells, and third, various (auto)immune suppressor-cytotoxic mechanisms, induced by the presence of abnormal or apoptotic hematopoietic stem cells or by the altered marrow microenvironment [8]. Regardless of the contributing pathogenetic mechanism(s), the result is gradual clonal dominance and expansion, thus leading to clinically active disease. MDS are currently classified, and prognostically categorized according to the severity of morphologic dysplasia (single lineage versus multilineage) the percentage of bone marrow blasts and the type of cytogenetic abnormalities. The most usually applied prognostic categorization system is the International Prognostic Scoring System (IPSS) in its initially reported version or in its revised version [9, 10]. From the management point of view, MDS patients are characterized as suffering from Lower- (IPSS ≤ 1 or IPSS-R ≤ 3.5) or Higher-risk disease (IPSS > 1 or IPSS-R > 3.5).

Currently, the major frontline treatment approach of the high-risk MDS and of elderly AML patients is the administration of hypomethylating agents (HMAs) such as 5-azacytidine, and 5-aza-2′-deoxycytidine (decitabine) [11]. As previously mentioned, epigenetic modulation has been shown to play a crucial role in the pathogenesis of several myeloid malignancies, including MDS and HMAs have been proven efficient toward treating such diseases [12]. Therefore, focusing on the identification of predictive epigenetic biomarkers for the response to HMA treatment remains a promising approach. Differences in methylation levels of *BCL2L10, EZH2, NOTCH1, CDA, CDKN2B* genes could affect the response of individuals with MDS/AML to AZA treatment [13].

Previous studies have identified candidate disease biomarkers, consisting of genomic loci involved in DNA methylation processes, CpG islands, or miRNAs. For example, it has been shown that epigenetic inactivation of *CTNNA1* and hypermethylation of 4-aminobutyrate aminotransferase (ABAT) could be associated with the progression of MDS to more aggressive subtypes or AML [14, 15]. A genome-wide methylation MethylCap-seq analysis on individuals with AML treated with decitabine showed significantly reduced methylation levels, thus suggesting a predicting tool for epigenetic-targeting therapies [16]. Moreover, other studies have focused on the role of miRs, such as miR-29b, miR-15a and miR-15b in HMA treatment response of individuals with MDS/AML and the pathogenesis of AML. With regard to the pathogenesis of AML, researchers have focused on evaluating the role of aberrant methylation of tumor suppressor genes (such as *CDKN2B*, *CDH13*, *GSTM5, RERG*)*.* In addition, the aberrant methylation profile of genes such as, *DLX5*, *SOX30*, *CDH1*, *p15(INK4B)* promoter, is significantly higher in AML and MDS-derived AML and could act as a predictive biomarker [17–21].

One of the recent methods for whole genome methylation analysis is based on DNA methylation-dependent restriction enzymes. Boers and collaborators showed that the activity of DNA methylation-dependent enzyme, LpnPI, is blocked by a fragment size smaller than 32 bp, preventing the complete digestion of methylation-dense DNA and thus allowing accurate genome-wide analysis of CpG methylation at single-nucleotide resolution [22]. This methodology has been already used to assess disease pathogenesis such as alveolar capillary dysplasia and desmoid-type fibromatosis [23, 24].

Here, we employed genome-wide methylation profiling in previously untreated individuals with higher-risk MDS or MDS/AML, who received HMA treatment, to assess how the aberrant methylation profile is involved in MDS progression to AML or to different MDS subtypes. To our knowledge, this is the first report of genome-wide assessment of methylation changes, using a high-throughput sequencing-based approach with the DNA methylation-dependent enzyme, LpnPI to agnostically assess the epigenomic basis of MDS pathogenesis and progression to AML and drug response.

## Materials and methods

### Individuals and samples

This pilot study included individuals who were diagnosed with previously untreated higher risk MDS according to World Health Organization 2016 classification, and who were treated with 5-azacitidine or decitabine. Overall, the present study included individuals diagnosed with RAEBI (n = 3), with RAEBII (n = 4) and individuals with MDS and multilineage dysplasia, but without an excess of blasts (n = 4). Moreover, the study included 11 individuals who had progressed to AML following a previous MDS. Of the total number of individuals (n = 22), 13 were treated with 5-azacitidine and 2 with decitabine. Among them, 6 individuals responded well, and 9 individuals were partial or non-responders to the HMA treatment (Additional file 1).

Informed consent was obtained by all study participants, while the study was approved by the Institutional Review Board of the Patras University General Hospital in Rion Patras, Greece (Approval document No. 33807/24.12.20). All the experiments involving human subjects were conducted according to the principles expressed in the Declaration of Helsinki.

Whole DNA was extracted from the bone marrow samples according to the phenol: chloroform DNA extraction method. The DNA concentration per analyzed sample was approximately 1000 ng/uL and of high purity. DNA concentration and purity were determined with NanoDrop spectrophotometer working on the principle of ultraviolet–visible spectrum (UV–Vis) absorbance (Quawell Q6000).

### Methylated DNA sequencing (MeD-seq)

#### Sample preparations

MeD-seq analyses were essentially carried out as previously described [22]. In brief, 22 DNA samples were digested by LpnPI (New England Biolabs, Ipswich, MA, USA), resulting in snippets of 32 bp around a fully methylated recognition site that contains a CpG. These short DNA fragments were further processed using the ThruPlex DNA–seq 96D kit (Rubicon Genomics Ann Arbor, MI, USA). Stem-loop adapters were blunt-end ligated to repair input DNA and amplified to include dual indexed barcodes using a high-fidelity polymerase to generate an indexed Illumina NGS library. The amplified end product was purified on a Pippin HT system with 3% agarose gel cassettes (Sage Science, Beverly, MA, USA). Multiplexed samples were sequenced on Illumina HiSeq2500 systems for single read of 50 bp according to manufacturer’s instructions. Dual indexed samples were de-multiplexed using bcl2fastq software (Illumina, San Diego, CA, USA).

### MeD-seq data analysis

Data processing was carried out using specifically created scripts in Python. Raw fastq files were subjected to Illumina adaptor trimming and reads were filtered based on LpnPI restriction site occurrence between 13 and 17 bp from either 5′ or 3′ end of the read. Reads that passed the filter were mapped to hg38 using bowtie2. Genome-wide individual LpnPI site scores were used to generate read count scores for the following annotated regions: transcription start sites (TSS, 1 kb before and 1 kb after), CpG-islands and gene bodies (1 kb after TSS until TES). Gene and CpG-island annotations were downloaded from ENSEMBL (www.ensembl.org). Detection of differentially methylated regions (DMRs) was performed between the datasets containing the regions of interest (TSS, gene body or CpG-islands) using the Chi-square test on read counts. Significance at a p-value of 0.05 was called by either Bonferroni or FDR using the Benjamini–Hochberg procedure [22].

In addition, a genome-wide sliding window was used to detect sequentially differentially methylated LpnPI sites. Statistical significance was called between LpnPI sites in predetermined groups using the Chi-square test. Neighboring significantly called LpnPI sites were binned and reported. Annotation of the overlap of genome-wide detected DMRs was reported for TSS, CpG-islands and gene body regions. DMR thresholds were based on LpnPI site count, DMR sizes (in bp) and fold changes of read counts as mentioned in the figure legends before performing hierarchical clustering. The differentially methylated datasets generated and analyzed during the current study have been deposited to the Sequence Read Archive (SRA) data repository (pending accession number).

Finally, and after taking into consideration the identified epigenetic targets with fold change above 3.5, an interaction map was constructed using PICKLE (accessed November 2021) and Cytoscape (version 3.9.0).

## Results

Only DMRs which had fold-changes ≥ 3.5 are included in Tables 1, 2, 3, 4, 5. These tables show the chromosomal start and end positions of the DMRs with a fold-change ≥ 3.5 and the overlapping genes associated with the DMR, the location of the DMR with respect to the gene body. Genome-wide individual LpnPI site scores were used to generate read count scores for the following annotated regions: transcription start sites [(TSS), 1 kb before and 1 kb after], CpG-islands and regions starting at 1 Kb after the TSS until the transcription end site (TES) thus corresponding to the gene body without promoter region [(postTSS1KB-TES)].

**Table 1:** Differentially methylated regions (DMRs) with fold change values above 3.5 after comparing extensive against partial responders to HMA treatment

Chromosome	Start	End	Fold Change	Genomic loci description	Genomic annotation
chr18	50,268,329	50,269,073	9.36	CpG-island	CpG22007
chr18	50,268,329	50,269,073	9.36	postTSS1KB-TES	*MBD1*
chr10	132,798,382	132,798,411	8.08	CpG-island	CpG6383
chr16	89,191,731	89,192,381	5.98	CpG-island	CpG18255
chr16	89,191,731	89,192,381	5.98	postTSS1KB-TES	*CDH15*
chr7	158,425,441	158,425,470	5.79	CpG-island	CpG47995
chr7	158,425,441	158,425,470	5.79	postTSS1KB-TES	*PTPRN2*
chr9	113,059,365	113,090,635	5.33	CpG-island	CpG51787
chr1	3,073,145	3,073,734	4.72	CpG-island	CpG275
chr1	3,073,145	3,073,734	4.72	postTSS1KB-TES	*PRDM16*
chr20	33,667,004	33,668,579	4.64	CpG-island	CpG30320
chr20	33,667,004	33,668,579	4.64	TSS	*ACTL10*
chr20	33,667,004	33,668,579	4.64	postTSS1KB-TES	*NECAB3*
chr20	33,667,004	33,668,579	4,64	postTSS1KB-TES	*ACTL10*
chr1	143,653,686	143,654,706	4,46	CpG-island	CpG2556
chr1	143,653,686	143,654,706	4,46	CpG-island	CpG2557
chr19	35,994,135	35,994,360	4,22	CpG-island	CpG24507
chr19	35,994,135	35,994,360	4,22	TSS	*SDHAF1*
chr19	7,862,503	7,862,711	4,08	CpG-island	CpG23476
chr19	7,862,503	7,862,711	4,08	postTSS1KB-TES	*EVI5L*
chr1	228,276,077	228,276,359	3,92	CpG-island	CpG4054
chr1	228,276,077	228,276,359	3,92	TSS	RP5-1139B12.3
chr1	228,276,077	228,276,359	3,92	postTSS1KB-TES	*OBSCN*
chr5	191,246	191,885	3,80	CpG-island	CpG38456
chr5	191,246	191,885	3,80	TSS	*LRRC14B*
chr1	2,122,668	2,122,914	3,65	CpG-island	CpG192
chr1	2,122,668	2,122,914	3,65	postTSS1KB-TES	*PRKCZ*
chr19	3,980,801	3,980,830	3,65	postTSS1KB-TES	*EEF2*
chr10	59,881,067	59,883,404	3,63	CpG-island	CpG5231
chr10	59,881,067	59,883,404	3,63	postTSS1KB-TES	*CCDC6*
chr19	58,356,134	58,356,892	3,59	CpG-island	CpG25795
chr19	58,356,134	58,356,892	3,59	postTSS1KB-TES	CTD-2619J13.8
chr19	58,356,134	58,356,892	3,59	postTSS1KB-TES	*ZNF497*

Chromosome loci of the DMRs, fold change and genomic annotation with regard to the identified DMRs. *TSS* Transcription Start Site, *TES* Transcription End Site; postTSS1KB-TES, indicates the region starting at 1 Kb after the TSS until the TES thus corresponding to the gene body without promoter region

**Table 2:** Differentially methylated regions (DMRs) with fold change values above 3.5 after comparing individuals with MDS who progressed to AML against individuals with MDS who did not

Chromosome	Start	End	Fold Change	Genomic loci description	Genomic annotation
chr6	169,966,466	169,968,275	9.52	CpG-island	CpG43837
chr1	247,170,879	247,172,787	5.79	CpG-island	CpG4368
chr1	247,170,879	247,172,787	5.79	TSS	*ZNF124*
chr1	247,170,879	247,172,787	5.79	postTSS1KB-TES	*ZNF124*
chr4	186,433,489	186,435,606	5.27	postTSS1KB-TES	*F11-AS1*
chr4	186,433,489	186,435,606	5.27	postTSS1KB-TES	*RP11-215A19.2*
chr10	133,292,034	133,292,157	5.04	CpG-island	CpG6468
chr10	133,292,034	133,292,157	5.04	postTSS1KB-TES	*TUBGCP2*
chr1	247,126,037	247,127,603	4.89	CpG-island	CpG4367
chr1	247,126,037	247,127,603	4.89	postTSS1KB-TES	*ZNF124*
chr10	75,405,211	75,405,359	3.73	CpG-island	CpG5460
chr10	75,405,211	75,405,359	3.73	postTSS1KB-TES	*ZNF503-AS2*

Chromosome loci of the DMRs, fold change and genomic annotation with regard to the identified DMRs. *TSS* Transcription Start Site; *TES* Transcription End Site; postTSS1KB-TES, indicates the region starting at 1 Kb after the TSS until the TES thus corresponding to the gene body without promoter region

**Table 3:** Differentially methylated regions (DMRs) with fold change values above 3.5 after comparing individuals diagnosed with RAEBI syndrome against individuals with RAEBII syndrome

Chromosome	Start	End	Fold Change	Genomic loci description	Genomic annotation
chr5	141,407,652	141,408,878	4.92	CpG-island	CpG40370
chr5	141,407,652	141,408,878	4.92	TSS	*PCDHGB6*
chr5	141,407,652	141,408,878	4.92	postTSS1KB-TES	*PCDHGA1*
chr5	141,407,652	141,408,878	4.92	postTSS1KB-TES	*PCDHGA2*
chr5	141,407,652	141,408,878	4.92	postTSS1KB-TES	*PCDHGA3*
chr5	141,407,652	141,408,878	4.92	postTSS1KB-TES	*PCDHGB1*
chr5	141,407,652	141,408,878	4.92	postTSS1KB-TES	*PCDHGA4*
chr5	141,407,652	141,408,878	4.92	postTSS1KB-TES	*PCDHGB2*
chr5	141,407,652	141,408,878	4.92	postTSS1KB-TES	*PCDHGA5*
chr5	141,407,652	141,408,878	4.92	postTSS1KB-TES	*PCDHGB3*
chr5	141,407,652	141,408,878	4.92	postTSS1KB-TES	*PCDHGA6*
chr5	141,407,652	141,408,878	4.92	postTSS1KB-TES	*PCDHGA7*
chr5	141,407,652	141,408,878	4.92	postTSS1KB-TES	*PCDHGB4*
chr5	141,407,652	141,408,878	4.92	postTSS1KB-TES	*PCDHGA8*
chr5	141,407,652	141,408,878	4.92	postTSS1KB-TES	*PCDHGB5*
chr5	141,407,652	141,408,878	4.92	postTSS1KB-TES	*PCDHGA9*
chr15	26,773,151	26,773,665	3.51	CpG-island	CpG14624
chr15	26,773,151	26,773,665	3.51	postTSS1KB-TES	*GABRB3*

Chromosome loci of the DMRs, fold change and genomic annotation with regard to the identified DMRs. *TSS* Transcription Start Site, *TES* Transcription End Site; postTSS1KB-TES, indicates the region starting at 1 Kb after the TSS until the TES thus corresponding to the gene body without promoter region

**Table 4:** Differentially methylated regions (DMRs) with fold change values above 3.5 after comparing individuals with RAEBI MDS subtype against individuals with MDS but without an excess of blasts

Chromosome	Start	End	Fold Change	Genomic loci description	Genomic annotation
chr5	141,491,020	141,491,136	5.70	postTSS1KB-TES	*PCDHGA1*
chr5	141,491,405	141,491,458	4.96	postTSS1KB-TES	*PCDHGA1*
chr5	141,491,020	141,491,136	5.70	postTSS1KB-TES	*PCDHGA2*
chr5	141,491,405	141,491,458	4.96	postTSS1KB-TES	*PCDHGA2*
chr5	141,491,020	141,491,136	5.70	postTSS1KB-TES	*PCDHGA3*
chr5	141,491,405	141,491,458	4.96	postTSS1KB-TES	*PCDHGA3*
chr5	141,491,020	141,491,136	5.70	postTSS1KB-TES	*PCDHGB1*
chr5	141,491,405	141,491,458	4.96	postTSS1KB-TES	*PCDHGB1*
chr5	141,491,020	141,491,136	5.70	postTSS1KB-TES	*PCDHGA4*
chr5	141,491,405	141,491,458	4.96	postTSS1KB-TES	*PCDHGA4*
chr5	141,491,020	141,491,136	5.70	postTSS1KB-TES	*PCDHGB2*
chr5	141,491,405	141,491,458	4.96	postTSS1KB-TES	*PCDHGB2*
chr5	141,491,020	141,491,136	5.70	postTSS1KB-TES	*PCDHGA5*
chr5	141,491,405	141,491,458	4.96	postTSS1KB-TES	*PCDHGA5*
chr5	141,491,020	141,491,136	5.70	postTSS1KB-TES	*PCDHGB3*
chr5	141,491,405	141,491,458	4.96	postTSS1KB-TES	*PCDHGB3*
chr5	141,491,020	141,491,136	5.70	postTSS1KB-TES	*PCDHGA6*
chr5	141,491,405	141,491,458	4.96	postTSS1KB-TES	*PCDHGA6*
chr5	141,491,020	141,491,136	5.70	postTSS1KB-TES	*PCDHGA7*
chr5	141,491,405	141,491,458	4.96	postTSS1KB-TES	*PCDHGA7*
chr5	141,491,020	141,491,136	5.70	postTSS1KB-TES	*PCDHGB4*
chr5	141,491,405	141,491,458	4.96	postTSS1KB-TES	*PCDHGB4*
chr5	141,491,020	141,491,136	5.70	postTSS1KB-TES	*PCDHGA8*
chr5	141,491,405	141,491,458	4.96	postTSS1KB-TES	*PCDHGA8*
chr5	141,491,020	141,491,136	5.70	postTSS1KB-TES	*PCDHGB5*
chr5	141,491,405	141,491,458	4.96	postTSS1KB-TES	*PCDHGB5*
chr5	141,491,020	141,491,136	5.70	postTSS1KB-TES	*PCDHGA9*
chr5	141,491,405	141,491,458	4.96	postTSS1KB-TES	*PCDHGA9*
chr5	141,491,020	141,491,136	5.70	postTSS1KB-TES	*PCDHGB6*
chr5	141,491,405	141,491,458	4.96	postTSS1KB-TES	*PCDHGB6*
chr15	26,891,159	26,892,850	4.89	CpG-island	CpG14631
chr15	26,891,159	26,892,850	4.89	postTSS1KB-TES	*GABRB3*
chr15	26,891,159	26,892,850	4.89	postTSS1KB-TES	*GABRA5*
chr15	26,773,368	26,773,480	3.74	CpG-island	*CpG14624*
chr15	26,773,368	26,773,480	3.74	postTSS1KB-TES	*GABRB3*

Chromosome loci of the DMRs, fold change and genomic annotation with regard to the identified DMRs. *TSS* Transcription Start Site, *TES* Transcription End Site; postTSS1KB-TES, indicates the region starting at 1 Kb after the TSS until the TES thus corresponding to the gene body without promoter region

**Table 5:** Differentially methylated regions (DMRs) with fold change values above 3.5 after comparing individuals with RAEBII MDS subtype against individuals with MDS but without an excess of blasts

Chromosome	Start	End	Fold Change	Genomic loci description	Genomic annotation
chr5	141,407,652	141,408,677	7.84	CpG-island	CpG40370
chr5	141,407,652	141,408,677	7.84	TSS	*PCDHGB6*
chr5	141,407,652	141,408,677	7.84	postTSS1KB-TES	*PCDHGA1*
chr5	141,508,267	141,508,909	4.76	postTSS1KB-TES	*PCDHGA1*
chr5	141,407,652	141,408,677	7.84	postTSS1KB-TES	*PCDHGA2*
chr5	141,508,267	141,508,909	4.76	postTSS1KB-TES	*PCDHGA2*
chr5	141,407,652	141,408,677	7.84	postTSS1KB-TES	*PCDHGA3*
chr5	141,508,267	141,508,909	4.76	postTSS1KB-TES	*PCDHGA3*
chr5	141,407,652	141,408,677	7.84	postTSS1KB-TES	*PCDHGB1*
chr5	141,508,267	141,508,909	4.76	postTSS1KB-TES	*PCDHGB1*
chr5	141,407,652	141,408,677	7.84	postTSS1KB-TES	*PCDHGA4*
chr5	141,508,267	141,508,909	4.76	postTSS1KB-TES	*PCDHGA4*
chr5	141,407,652	141,408,677	7.84	postTSS1KB-TES	*PCDHGB2*
chr5	141,508,267	141,508,909	4.76	postTSS1KB-TES	*PCDHGB2*
chr5	141,407,652	141,408,677	7.84	postTSS1KB-TES	*PCDHGA5*
chr5	141,508,267	141,508,909	4.76	postTSS1KB-TES	*PCDHGA5*
chr5	141,407,652	141,408,677	7.84	postTSS1KB-TES	*PCDHGB3*
chr5	141,508,267	141,508,909	4.76	postTSS1KB-TES	*PCDHGB3*
chr5	141,407,652	141,408,677	7.84	postTSS1KB-TES	*PCDHGA6*
chr5	141,508,267	141,508,909	4.76	postTSS1KB-TES	*PCDHGA6*
chr5	141,407,652	141,408,677	7.84	postTSS1KB-TES	*PCDHGA7*
chr5	141,508,267	141,508,909	4.76	postTSS1KB-TES	*PCDHGA7*
chr5	141,407,652	141,408,677	7.84	postTSS1KB-TES	*PCDHGB4*
chr5	141,508,267	141,508,909	4.76	postTSS1KB-TES	*PCDHGB4*
chr5	141,407,652	141,408,677	7.84	postTSS1KB-TES	*PCDHGA8*
chr5	141,508,267	141,508,909	4.76	postTSS1KB-TES	*PCDHGA8*
chr5	141,407,652	141,408,677	7.84	postTSS1KB-TES	*PCDHGB5*
chr5	141,508,267	141,508,909	4.76	postTSS1KB-TES	*PCDHGB5*
chr5	141,407,652	141,408,677	7.84	postTSS1KB-TES	*PCDHGA9*
chr5	141,508,267	141,508,909	4.76	postTSS1KB-TES	*PCDHGA9*
chr5	141,508,267	141,508,909	4.76	postTSS1KB-TES	*PCDHGB6*
chr15	26,891,832	26,892,546	4.31	CpG-island	CpG14631
chr15	26,891,832	26,892,546	4.31	postTSS1KB-TES	*GABRB3*
chr15	26,891,832	26,892,546	4.31	postTSS1KB-TES	*GABRA5*

Chromosome loci of the DMRs, fold change and genomic annotation with regard to the identified DMRs. *TSS* Transcription Start Site, *TES* Transcription End Site; postTSS1KB-TES, indicates the region starting at 1 Kb after the TSS until the TES thus corresponding to the gene body without promoter region

### Differences in the methylome profile between good and partial HMA treatment responders

We assessed whole-genome methylation data and searched for differences in the methylome profiles between good (n = 6) and partial (n = 9) responders to HMA treatment. The main criterion in this sub-analysis was the fold change value, which was used to pinpoint differentially methylated regions (DMRs) regardless of whether they are hypo- or hyper-methylated in a certain group. Among the targets with the most notable changes in terms of methylation fold change (i.e., fold change value above 5) were DMRs within the following CpG islands, namely CpG22007, CpG6383 and CpG18255, located within chromosomes 18, 10 and 16, respectively. Moreover, DMRs within the chromosomes 18, 16 and 7 were also found to significantly affect the transcription of certain genes, including *MBD1, CDH15* and *PTPRN2* (Table 1)*.*

**Fig. 1:**
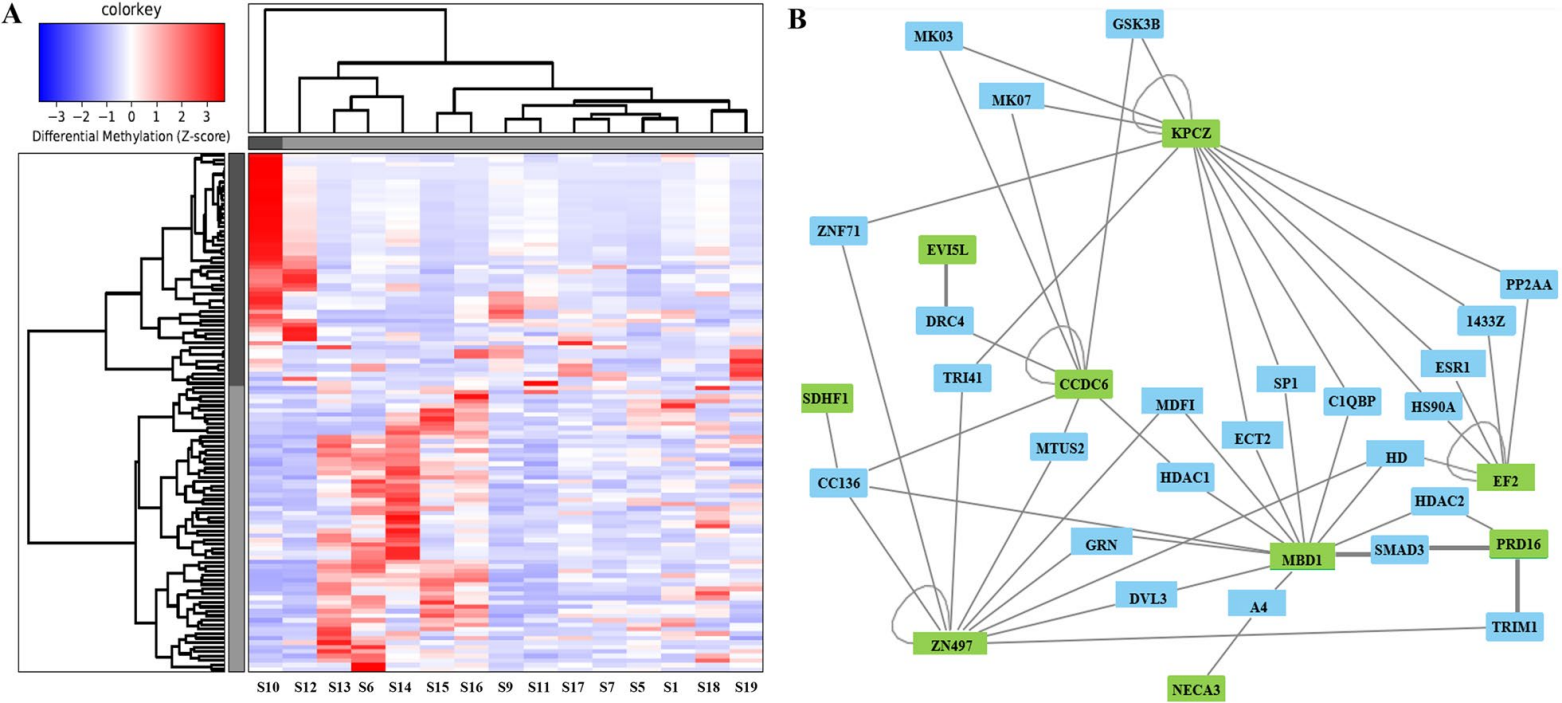
Heatmap depicting DMRs upon comparing good and partial HMA treatment responders (**A**). Areas hypomethylated are depicted with blue and areas hypermethylated are depicted with red. Network map (**B**) depicting interactions between DMRs with fold change values above 3.5 and other proteins involved in molecular pathways. These DMRs are also found within the heatmap (**A**). Good ΗΜΑ treatment responder IDs: S10, S12, S9, S17, S7, S19; Partial ΗΜΑ treatment responder IDs: S13, S6, S14, S15, S16, S11, S5, S1, S18

Among our interesting observations was the DMR found within the gene body of *ZNF497* (Fig. 1A). As also depicted in the interaction map, an interaction of *ZNF497* gene with different HDAC enzymes (histone deacetylase enzymes) using cytoscape was observed and which could be involved in epigenetic regulation processes (Fig. 1B).

### Differences in the methylome profile for individuals with MDS progressing to AML

**Fig. 2:**
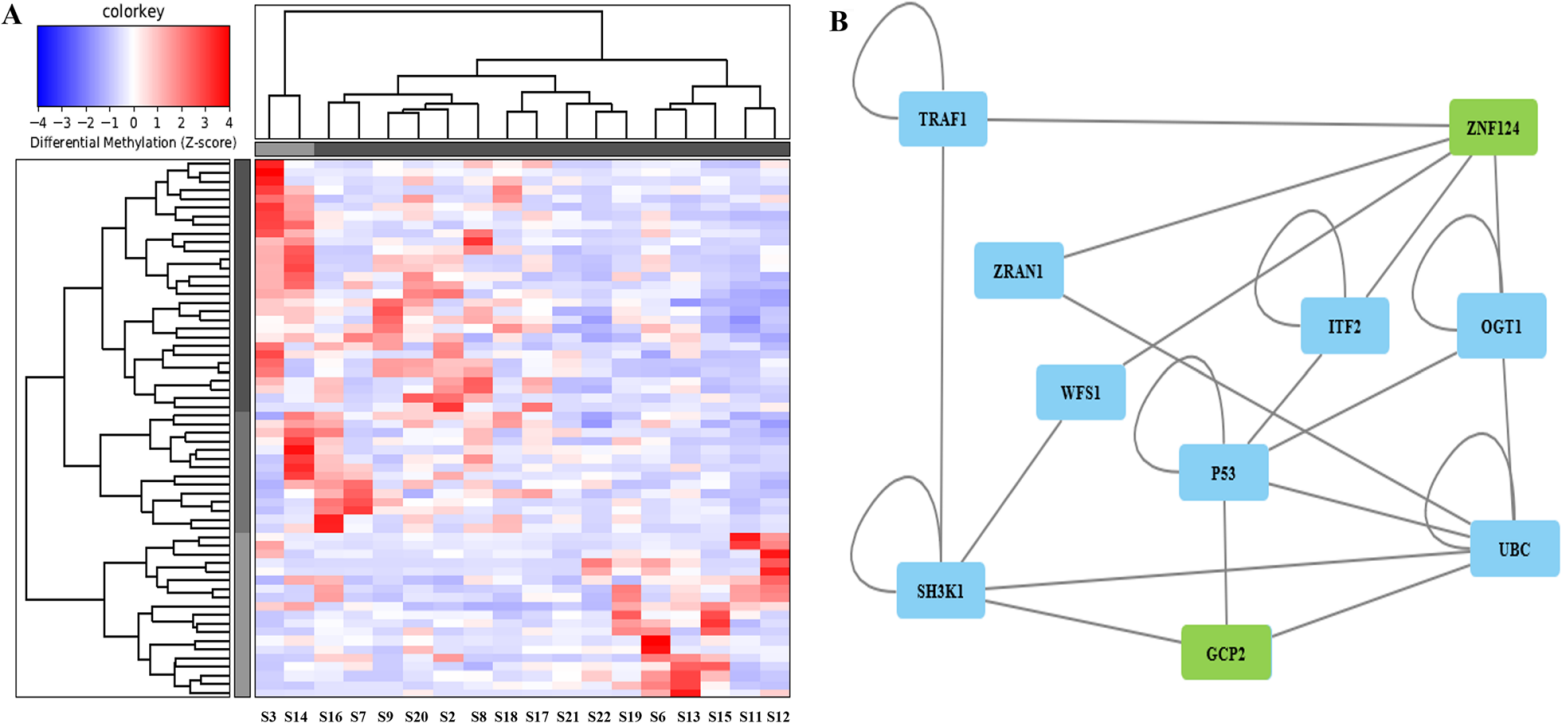
Heatmap depicting DMRs upon comparing individuals with MDS who progressed to AML against those who did not (**A**). Areas hypomethylated are depicted with blue and areas hypermethylated are depicted with red. Network map (**B**) depicting interactions between DMRs with fold change values above 3.5 and other proteins involved in molecular pathways. These DMRs are also found within the heatmap (**A**). Progress to aggressive AML: S3, S14, S16, S7, S9, S20, S2, S8, S18, S17, S21; Without progress to aggressive AML: S22, S19, S6, S13, S15, S11, S12

Next, we assessed for differences in the methylation profiles of individuals diagnosed with MDS and who may or may not have progressed to AML. Among our findings were DMRs with high fold change values that were observed with the following CpG islands, namely CpG43837 and CpG4368. The latter DMR overlaps with a CpG island (CpG4368), the TSS and the gene body region of *ZNF124.* Interestingly, the majority of the identified DMRs, stemming from this comparison, included DMRs within chromosomes 1 and 10 and were located similarly in gene body regions of Zinc finger family genes, the differential methylation of which could be involved in epigenetic processes (Table 2, Fig. 2A). As also shown in Fig. 2B, there is a potential interaction of *ZN124* gene with GCP2 gene via p53 protein. Moreover, *GCP2*, which is a component of the gamma tubulin complex, is heavily involved in microtubule nucleation.

### Differences in the methylome profile between individuals diagnosed with an excess of marrow blasts (RAEBI or RAEBII)

**Fig. 3:**
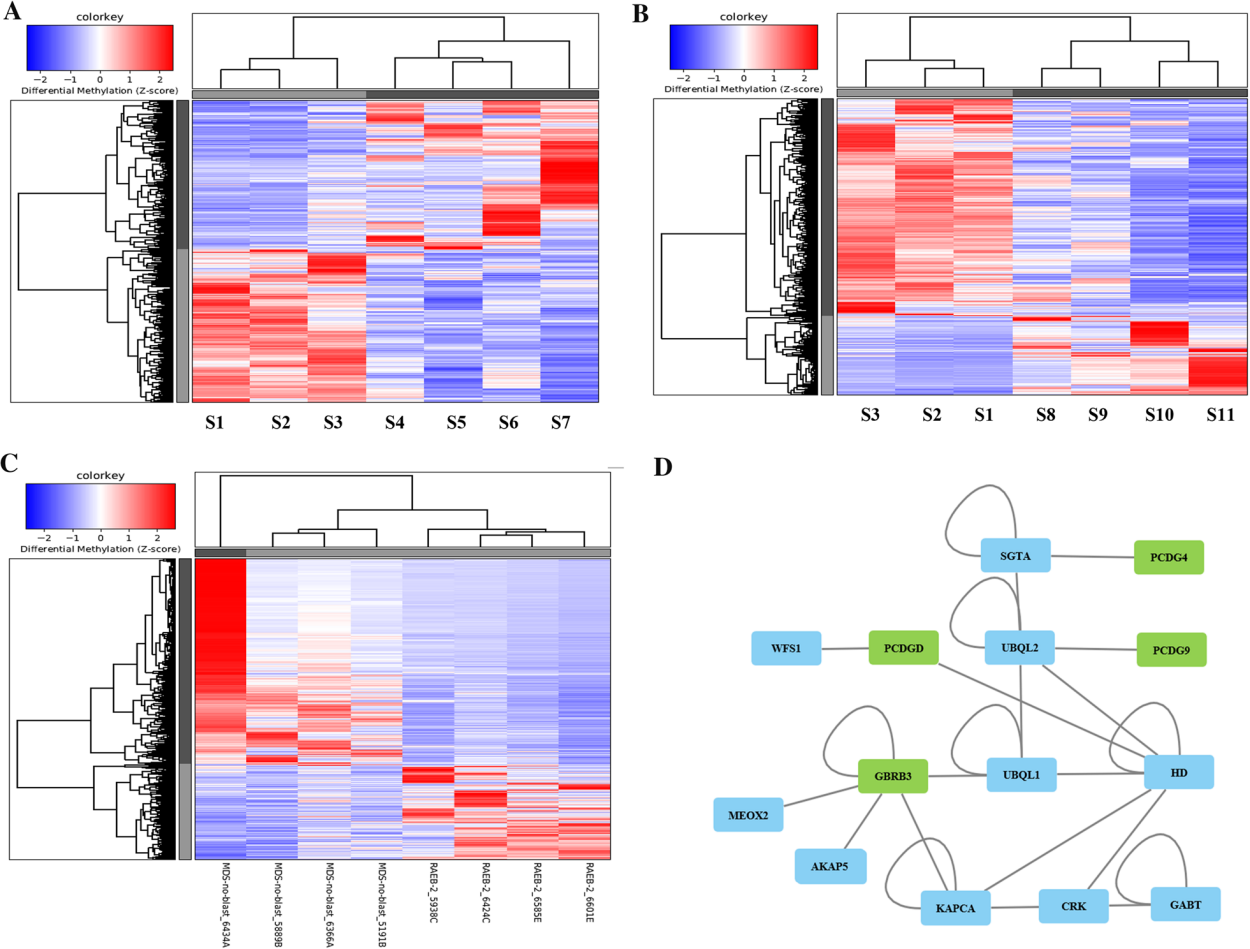
Heatmap depicting DMRs upon comparing individuals with RAEBI MDS subtype against individuals with MDS but without an excess of blasts (**A**). Another heatmap depicting DMRs upon comparing individuals with RAEBI MDS subtype against individuals with MDS but without an excess of blasts (**B**). Heatmap depicting DMRs upon comparing individuals with RAEBII MDS subtype against individuals with RAEBII MDS subtype (**C**). Network map (**D**) depicting interactions between DMRs with fold change values above 3.5 and other proteins involved in molecular pathways. These DMRs are also found within the heatmaps (**A**, **B**, **C**). RAEBI: S1, S2, S3; RAEBII: S4, S5, S6, S7; MDS without an excess of blasts: S8, S9, S10, S11

After comparing individuals diagnosed with RAEBI MDS subtype against individuals diagnosed with RAEBII MDS subtype, we obtained good separation between both groups. Specifically, genetic targets hypomethylated in individuals with RAEBII MDS subtype were hypermethylated in RAEBI individuals and vice versa (Fig. 3A). Among the targets with the most notable methylation fold change were DMRs found exclusively within the *PCDHG* gene family in the gene body methylation of these genes (Table 3**, **Fig. 3A).

### Differences in the methylome profile between individuals with RAEBI MDS subtype and individuals without an excess of blasts

After comparing individuals with RAEBI MDS subtype against individuals without an excess of blasts, methylation differences were observed that distinguished both groups. Specifically, genetic targets hypomethylated in individuals with RAEBI MDS subtype were hypermethylated in individuals without an excess of blasts and vice versa (Fig. 3B). The DMRs with the most significant changes in terms of methylation fold change were again located in gene body regions of the *PCDHGs* (Table 3).

### Differences in the methylome profile between RAEBII and samples without an excess of blasts

After comparing individuals with RAEBII MDS subtype against individuals diagnosed with MDS but without an excess of blasts, a very clear differential methylation pattern was once again identified. Similar to previous comparisons, DMRs hypomethylated in individuals with RAEBII MDS subtype were hypermethylated in samples without an excess of blasts and vice versa **(**Fig. 3C**)**. Of note, DMRs with the most significant changes in terms of methylation fold change were again observed in the *PCDHG* gene family with fold change values above 3.5 **(**Table 3**)**.

According to findings from “Differences in the methylome profile between good and partial HMA treatment responders,” “Differences in the methylome profile for individuals with MDS progressing to AML,” “Differences in the methylome profile between individuals diagnosed with an excess of marrow blasts (RAEBI or RAEBII)” sections and the interaction map, it is depicted that *GABRB3*, which harbors DMRs with notable fold change values, potentially interacts with different genes from the *PCDHG* gene family** (**Fig. 3D).

## Discussion

Over the last decade, high throughput sequencing technologies have assisted toward unravelling the genomic and epigenomic background of many diseases. Herein, we utilized whole methylome sequencing to identify DMRs within individuals with myelodysplastic syndromes.

Among the identified targets are DMRs located within the *PCDHG* gene family. Protocadherins consist of a subfamily of the cadherin gene family and play a significant role in the development of the nervous system, thus being involved in pathways for cell proliferation and death pathways. To the best of our knowledge, the available literature on PCDH and the current studies associating the PCDH methylation levels with the development of different types of cancer is limited [25].

*PCDHGC3* has been indicated as a potential biomarker, to identify individuals with paragangliomas and pheochromocytomas with an increased risk of metastasis [26]. Moreover, according to Dallosso and coworkers, 15 out of 19 tested *PCDHG* genes were affected by an aberrant methylation detected in all subtypes of Wilms' tumor [27]. Specifically, the aberrant *PCDH10* methylation has been suggested as a prognostic biomarker in tumors [28, 29]. *PCDH17* gene was silenced by DNA methylation in AML and low *PCDH17* expression due to aberrant methylation grade was associated with improved risk stratification in individuals with AML [30].

Our study is the first—to the best of our knowledge—that reports the possible involvement of *PCDHG* and *GABA* methylation in the pathogenesis of myelodysplastic syndrome. Several signaling pathways were linked to the regulation of proliferation (Wnt/β-catenin signaling and Pi3K/AKT-signaling) and apoptosis (NF-κB and DEPDC1-caspase signaling) by PCDHs in cancer [31–39].

Our results further support this finding by demonstrating that the methylation profile of *PCDHGs* plays a crucial role in the pathophysiology of different types of MDS syndromes. The pathway involved and regulated by *PCDH* could be either Wnt signaling, the stabilization and maintenance of some GABAergic synapses or other signaling pathways or other stabilization.

Based on the results of our study, we suggest that the aberrant methylation level of *GABRA3* (gamma-aminobutyric acid (GABA) A receptor, subunit beta 3) and *GABRA5* (Gamma-Aminobutyric Acid Type A Receptor Subunit Alpha5) could play an important role in the MDS pathophysiology due to their impact through the GABAergic synapses. Among the 20 genes which have been investigated as prognostic biomarkers for AML is *GABRG3*, the mutated genes examined, seem to participate in several cancer pathways, including the PI3K/AKT and RAS/MAPK pathways [40]. Moreover, the p38 MAPK pathway has been mentioned as a key driver in the pathogenesis of MDS. The above results support the present outcomes of our study and the message hidden back from the highly altered methylome change of *GABRG3* in all the MDS types (RAEBI, RAEBII, and MDS without excess blasts). *ABAT* gene, which encodes a protein responsible for the catabolism of γ-aminobutyric acid (GABA), was both downregulated in MDS samples and cell lines. Moreover, Zhao and co-workers hypothesized that genomic variants in the *ABAT* gene may be involved in the pathogenesis of MDS, due to their correlation with the TCA (tricarboxylic acid cycle) cycle [15]. Taken together the GABA pathway could play a crucial role in the pathogenesis of MDS subtypes.

Based on our findings, the highly differentiated methylome profile of *GCP2* and its linkage with p53 could affect the MDS progression to AML. Oka and coworkers concluded that there is not any relationship between p53 expression, CD13/CD33 ratio, and the outcomes of MDS patients treated with AZA. Based on this study, a number of p53 variants affect p53 expression levels but not the overall survival and the AML progression. However, qualitative parameters in the expression of p53 including the functional integrity of the produced protein have not been taken into account. There are not any probable secondary pathways that might be activated or deactivated by the primary studied interactions and result in considerable alterations of the expected outcome. Consequently, the lack of observed differentiation in the expression of p53 in quantitative terms does not negatively inform our identification of such associations [41]. Similar to our findings, Dráberová and coworkers showed that dysregulation of γ-tubulin proteins in glioblastomas could be linked to a possible interaction with signaling pathways and hence lead to a malignant phenotype [42]. Overall, γ-tubulin is considered as a potential target to reduce tumor growth in a variety of malignancies.

We also reported DMRs with a significant fold change values located within ZNF family genes, which were associated with progression to AML and with response to HMA treatment. This observation is in line with previous studies, reporting that several KRAB-ZNFs could affect the expression as tumor suppressors in cell culture models, including *p53*, *MDM2*, *BRCA1* [43–45]. By comparing the methylome profile between MDS and healthy individuals, ZNF plays crucial role in repressive H3K9me3 modification, which demonstrates differential pattern among the individuals and the HDAC inhibition seems to affect terminal differentiation of myeloid tumor cells [46, 47].

## Conclusion

Our study demonstrated the role of ZNF transcription factors both in MDS progression to AML and in the response to HMA treatment. Furthermore, the role of *PCDHG* gene family seems to be crucial in the pathogenesis of MDS regardless of the MDS subtype. To the best of our knowledge, this is the first study that combines the (a) epigenomic role of aberrant methylation profile in MDS pathogenesis with progress to AML and (b) pharmacoepigenomic basis to the HMA drug treatment response. This agnostic genome-wide whole methylome analysis can be readily replicated to identify the epigenomic component of other genetic diseases, while further methylome studies are needed to better clarify the role of methylation profile in MDS pathogenesis and HMA response, which could lead to a better quality of life and survival.

## Supplementary Information

**Additional file 1.** Summary table of clinical information with regards to the samples. Information about the sample number, the diagnosis after biopsy, the Hypomethylating agents (HMA) treatment and the possible progression to Acute Myeloid Leukemia (AML) is shown.

## Abbreviations

ABAT: 4-Aminobutyrate aminotransferase
AML: Acute myeloid leukemia
DMRs: DMRs differentially methylated regions
HMAs: Hypomethylating agents
MeD-seq: Methylated DNA sequencing
MDS: Myelodysplastic syndromes
RAEB I: Refractory anemia with excess blasts I
RAEBII: Refractory anemia with excess blasts II
TES: Transcription end site
TSS: Transcription start sites
ZNF: Zinc-finger
