# Supplementary material for: Genome-wide analysis toward the epigenetic aetiology of myelodysplastic syndrome disease progression and pharmacoepigenomic basis of hypomethylating agents drug treatment response
Source: Hum Genomics. 2023 Apr 25;17:37. doi: 10.1186/s40246-023-00483-7 (PMC10127336; doi:10.1186/s40246-023-00483-7)
Supplement: Supplementary file 1 — Additional file 1. Summary table of clinical information with regards to the samples. Information about the sample number, the diagnosis after biopsy, the Hypomethylating agents (HMA) treatment and the possible progression to Acute Myeloid Leukemia (AML) is shown. [file 40246_2023_483_MOESM1_ESM.docx]

**Supplementary information**

**Genome-wide analysis towards the epigenetic aetiology of myelodysplastic syndrome disease progression and pharmacoepigenomic basis of hypomethylating agents drug treatment response**

Stavroula Siamoglou ^1,#^, Ruben Boers ^2,#^, Maria Koromina ^1^, Joachim Boers ^2^, Anna Tsironi ^1^, Theodora Chatzilygeroudi ^3^, Vasileios Lazaris ^3^, Evgenia Verigou ^3^, Alexandra Kourakli ^3^, [Wilfred F J van IJcken](https://pubmed.ncbi.nlm.nih.gov/?term=van+IJcken+WFJ&cauthor_id=33194643) ^4^, Joost Gribnau ^2^ , Argiris Symeonidis ^3^, George P. Patrinos ^1,5,6^*

^1^ University of Patras, School of Health Sciences, Department of Pharmacy, Laboratory of Pharmacogenomics and Individualized Therapy, Patras, Greece

^2^ Erasmus Medical Center, Department of Developmental Biology, Rotterdam, the Netherlands

^3^ Hematology Division, Dept of Int. Medicine, University of Patras Medical School, Patras, Greece

^4^ Erasmus Medical Center, Center for Biomics, Rotterdam, The Netherlands

^5^ United Arab Emirates University, College of Medicine and Health Sciences, Department of Genetics and Genomics, Al-Ain, Abu Dhabi, United Arab Emirates

^6^ United Arab Emirates University, Zayed Center for Health Sciences, Al-Ain, Abu Dhabi, United Arab Emirates

^#^ These authors contributed equally to this work

**Supplementary Table 1.**

Summary table of clinical information with regards to the samples used. Information about the sample number, the diagnosis after biopsy, the Hypomethylating agents (HMA) treatment and the possible progression to Acute Myeloid Leukemia (AML) is shown. Abbreviations: RAEB Ι, Refractory anemia with excess blasts Ι; RAEBΙΙ, Refractory anemia with excess blasts ΙΙ. N/A: Not available.

| **Sample IDS** | **Type of MDS** | **HMA given** | **Progression to AML** |
| --- | --- | --- | --- |
| **3.1. Differences in the methylome profile between RAEB1 and RAEB2 sample** | | | |
| S1 | RAEB I | 5-azacytidine | Yes |
| S2 | RAEB I | 5-azacytidine | Yes |
| S3 | RAEBI | 5-azacytidine | Yes |
| S4 | RAEBII | 5-azacytidine | Yes |
| S5 | RAEBII | 5-aza-2′-deoxycytidine | Yes |
| S6 | RAEB II | 5-azacytidine | N/A |
| S7 | RAEBII | 5-azacytidine | N/A |
| **3.2. Differences in the methylome profile between RAEB1 and samples without an excess of blasts** | | | |
| S3 | RAEBI | 5-azacytidine | Yes |
| S2 | RAEB I | 5-azacytidine | Yes |
| S1 | RAEB I | 5-azacytidine | Yes |
| S8 | MDS without excess of blasts | 5-azacytidine | Yes |
| S9 | MDS without excess of blasts | 5-azacytidine | Yes |
| S10 | RAEBI | 5-azacytidine | Yes |
| S11 | MDS without excess of blasts | 5-azacytidine | No |
| **3.3. Differences in the methylome profile between RAEB2 and samples without an excess of blasts** | | | |
| S10 | MDS without excess of blasts | 5-azacytidine | Yes |
| S11 | MDS without excess of blasts | 5-azacytidine | No |
| S8 | MDS without excess of blasts | 5-azacytidine | Yes |
| S9 | MDS without excess of blasts | 5-azacytidine | Yes |
| S7 | RAEBII | 5-azacytidine | N/A |
| S6 | RAEBII | 5-azacytidine | N/A |
| S4 | RAEBII | 5-azacytidine | Yes |
| S5 | RAEBII | 5-aza-2′-deoxycytidine | Yes |
| **3.4. Differences in the methylome profile between good and partial HMA treatment responders** | | | |
| S10 | RAEBI | 5-azacytidine  Good Response | Yes |
| S12 | MDS without excess of blasts | 5-azacytidine  Good Response | N/A |
| S13 | MDS without excess of blasts | 5-azacytidine  Partial Response | N/A |
| S6 | RAEB II | 5-azacytidine  Partial Response | N/A |
| S14 | RAEB II | 5-azacytidine  Partial Response | Yes |
| S15 | MDS without excess of blasts | 5-azacytidine  Partial Response | No |
| S16 | RAEBII | 5-azacytidine  Partial Response | Yes |
| S9 | MDS without excess of blasts | 5-azacytidine  Good Response | Yes |
| S11 | MDS without excess of blasts | 5-azacytidine  Partial Response | No |
| S17 | RAEB II | 5-aza-2′-deoxycytidine  Good response | Yes |
| S7 | RAEBII | 5-azacytidine  Good Response | N/A |
| S5 | RAEBII | 5-aza-2′-deoxycytidine  Partial Response | Yes |
| S1 | RAEB I | 5-azacytidine  Partial Response | Yes |
| S18 | RAEBII | 5-azacytidine  Partial Response | Yes |
| S19 | RAEB II | 5-azacytidine  Good Response | NO |
| **3.5. Differences in the methylome profile for individuals progressing to AML** | | | |
| S3 | RAEBI | 5-azacytidine | Yes |
| S14 | RAEB II | 5-azacytidine | Yes |
| S16 | RAEBII | 5-azacytidine | Yes |
| S7 | RAEBII | 5-azacytidine | N/A |
| S9 | MDS without excess of blasts | 5-azacytidine | Yes |
| S20 | RAEB II | 5-aza-2′-deoxycytidine | Yes |
| S2 | RAEB I | 5-azacytidine | Yes |
| S8 | MDS without excess of blasts | 5-azacytidine | Yes |
| S18 | RAEBII | 5-azacytidine | Yes |
| S17 | RAEB II | 5-aza-2′-deoxycytidine | Yes |
| S21 | RAEB II | 5-aza-2′-deoxycytidine | Yes |
| S22 | RAEB II | 5-azacytidine | N/A |
| S19 | RAEB II | 5-azacytidine | No |
| S6 | RAEB II | 5-azacytidine | N/A |
| S13 | MDS without excess of blasts | 5-azacytidine | N/A |
| S15 | MDS without excess of blasts | 5-azacytidine | No |
| S11 | MDS without excess of blasts | 5-azacytidine | No |
| S12 | MDS without excess of blasts | 5-azacytidine | N/A |
